# Anti-CD19-Therapien: der kommende Goldstandard für die IgG4-assoziierten Erkrankungen

**DOI:** 10.1007/s00393-025-01682-4

**Published:** 2025-07-31

**Authors:** Martin Krusche

**Affiliations:** Sektion für Rheumatologie und Entzündliche Systemerkrankungen in der III. Medizin, Universitätsklinikum Hamburg-Eppendorf (UKE), Martinistr. 52, 20246 Hamburg, Deutschland

**Keywords:** B-Zelle, Plasmazelle, IgG4-Syndrom, Anti-CD19, B cell, Plasma cell, IgG4-RD, Anti CD19

## Abstract

Die IgG4-assoziierte Erkrankung (IgG4-RD) ist eine immunvermittelte, entzündlich fibrosierende Multiorganerkrankung. B‑ und Plasmazellen spielen eine zentrale Rolle in der Pathophysiologie, was B‑Zell-gerichtete Therapien besonders interessant für die Erkrankung macht. Während Glukokortikoide weiterhin die Erstlinientherapie darstellen, sind Rezidive unter Dosisreduktion häufig. Immunsuppressiva wie Methotrexat, Azathioprin, Mycophenolat-Mofetil oder Rituximab (Anti-CD20) werden eingesetzt, jedoch fehlen größere Studien zum Nutzennachweis, sodass bisher keine zugelassenen Therapieoptionen für die Erkrankung existieren. CD19 als therapeutisches Ziel adressiert ein breiteres Spektrum der B‑Zell-Differenzierung einschließlich Plasmablasten. Die multizentrische, randomisierte, placebokontrollierte Phase-3-Studie MITIGATE (*n* = 135) untersuchte erstmals den Anti-CD19-Antikörper Inebilizumab bei aktiver IgG4-RD. Die Behandlung reduzierte signifikant das Rezidivrisiko (10 % vs. 60 % unter Placebo), die jährliche Schubrate sowie die Notwendigkeit für erneute Glukokortikoidgaben. Zudem zeigten sich unter Inebilizumab eine mediane Reduktion der IgG4-Serumspiegel um 50 % sowie eine anhaltende B‑Zell-Depletion über die Studiendauer von 52 Wochen. Schwerwiegende unerwünschte Ereignisse traten häufiger unter Inebilizumab auf, insbesondere Infektionen und Lymphopenien, jedoch ohne therapieassoziierte Todesfälle. Nach der FDA-Zulassung von Inebilizumab für IgG-RD im April 2025 ist eine Zulassung zeitnah auch in Europa zu erwarten. Die Anti-CD19 gerichtete Therapie mit Inebilizumab könnte somit zum neuen zugelassenen Goldstandard werden. Langzeitdaten zur Remissionserhaltung sowie gesundheitsökonomische Fragestellungen, insbesondere im Vergleich zu Off-Label-Therapien wie Rituximab, bleiben jedoch Gegenstand weiterer Forschung. Neue Ansätze wie Anti-CD19-CAR-T-Zell-Therapien oder bispezifische T‑Zell-Engager könnten darüber hinaus künftig zusätzliche Behandlungsoptionen eröffnen.

## Abstract (en)

Immunoglobulin G4-related disease (IgG4-RD) is an immune-mediated, inflammatory, fibrosing multiorgan disease. The B cells and plasma cells play a central role in the pathophysiology, which makes B cell-targeted treatment particularly interesting for the disease. Although glucocorticoids still represent the first line treatment, recurrence under dose reduction is frequent. Immunosuppressants, such as methotrexate, azathioprine, mycophenolate mofetil or rituximab (anti-CD20) are used but larger studies on proof of benefits are lacking so that no approved treatment options for the disease currently exist. Using CD19 as the therapeutic target addresses a broad spectrum of B cell differentiation including plasmablasts. The multicenter randomized placebo-controlled phase 3 study MITIGATE (*n* = 135) first investigated the anti-CD19 antibody inebilizumab in active IgG4-RD. The treatment significantly reduced the risk of recurrence (10% vs. 60% under placebo), the annual exacerbation rate and the necessity for renewed administration of glucocorticoids. Additionally, under inebilizumab there was a median reduction of the IgG4 serum level of around 50% and a persisting depletion of B cells during the study period of 52 weeks. Severe undesired events occurred more frequently with inebilizumab, particularly infections and lymphopenia but without treatment-associated cases of death. Following approval of inebilizumab by the U.S. Food and Drug Administration (FDA) for IgG4-RD in 2025, approval in Europe is also soon to be expected. The anti-CD19 targeted treatment with inebilizumab could therefore become the new approved gold standard; however, long-term data on remission maintenance and health economic issues, especially in comparison to off-label treatment such as rituximab remain the subject of further research. New approaches, such as CD19 chimeric antigen receptor (CAR) T cell treatment or bispecific T cell engagers could furthermore open up future treatment options.

Seitdem im Jahr 2001 die Ig(Immunglobulin)G4-assoziierte Erkrankung („IgG4-related disease“ [IgG4-RD]) als eigenständige Erkrankungsentität definiert wurde, konnte in den letzten Jahren eine Vielzahl an experimentellen und klinischen Studien Fortschritte für das Verständnis dieser entzündlich fibrosierenden Multisystemerkrankung erzielen.

Pathophysiologisch weiß man heute, dass ein Zusammenspiel aus B‑Zellen, IgG4^+^-Plasmazellen, follikulären T‑Helfer-Zellen, zytotoxischen CD4^+^-T-Zellen und M2-Makrophagen eine Entzündungsreaktion mit profibrotischem Zytokinmilieu induzieren, die Fibroblasten zur Sekretion extrazellulärer Matrixkomponenten stimulieren. Histologisch zeigen sich bei der Erkrankung die charakteristischen Befunde einer lymphozytären Infiltration (IgG4/IgG-Ratio > 40 %) mit storiformer Fibrose und obliterativer Phlebitis, welche sich makroskopisch in einer diffusen Organvergrößerung oder in tumorösem Wachstum widerspiegeln [1].

Auch wenn 2019 erstmalig Klassifikationskriterien für die Erkrankung definiert wurden [2], existieren bis heute weder zugelassene Medikamente noch einheitlich validierte Therapiestandards für die Behandlung der IgG4-RD. In der klinischen Praxis werden primär Glukokortikoide eingesetzt. Allerdings treten unter Dosisreduktion oder Absetzen der Steroidtherapie häufig Krankheitsrezidive auf [3]. Als steroidsparende Strategien kommen daher verschiedene immunsuppressive Substanzen wie Methotrexat, Azathioprin, Mycophenolat-Mofetil (MMF) oder Rituximab [4] zum Einsatz. Für diese Substanzen – mit Ausnahme von MMF [5] – fehlen bislang jedoch größere prospektive, randomisiert kontrollierte Studien mit belastbarem Wirksamkeitsnachweis. Vor dem Hintergrund der zentralen Rolle von B‑ und Plasmazellen in der Pathophysiologie der Erkrankung erscheint deren therapeutische Adressierung besonders vielversprechend. Für die CD20-gerichtete Therapie bei IgG4-RD mit Rituximab liegen Registerdaten [6] sowie eine Open-label-Studie [7] vor, die auf einen klinischen Nutzen hinweisen. Eine Zulassung für die Behandlung der IgG4-RD liegt jedoch für Rituximab nicht vor.

Neben CD20 ist CD19 ein weiterer therapeutischer Angriffspunkt bei IgG4-RD, da die pathologischen Gewebeinfiltrate aus einer dichten lymphoplasmazellulären Infiltration bestehen, die reich an IgG4-positiven Plasmazellen und deren Vorläuferzellen ist. CD19 wird auf der gesamten B‑Zell-Linie exprimiert, einschließlich naiver B‑Zellen, Gedächtnis-B-Zellen und insbesondere Plasmablasten, die im aktiven Stadium der Erkrankung stark vermehrt auftreten. Im Jahr 2023 konnte erstmals eine Phase-2-Open-label Studie an 15 Patient:innen ein positives Therapieansprechen mit Obexelimab, einem monoklonalen Antikörper, der an CD19 und den Fc-Gamma-Rezeptor IIb bindet, bei IgG4-RD zeigen [8].

In der kürzlich im *New England Journal of Medicine* veröffentlichten MITIGATE-Studie wurde Inebilizumab (Handelsname Uplizna) zur Behandlung der IgG4-RD untersucht [9]. Bei Inebilizumab handelt es sich um einen monoklonalen Antikörper gegen CD19, der eine rasche und tiefgreifende B‑Zell-Depletion bewirken soll und bereits für die Behandlung der Neuromyelitis-optica-Spektrum-Erkrankungen (NMOSD) [10] erfolgreich eingesetzt wird (Zulassung 2022).

Die multizentrische, doppelblinde, placebokontrollierte Phase-3-Studie schloss 135 Patient:innen mit aktiver IgG4-RD ein und wurde an 80 Zentren in 22 Ländern durchgeführt. Einschlusskriterien waren ein Alter über 18 Jahre, eine Diagnosestellung gemäß den ACR/EULAR-Kriterien sowie mindestens 2 betroffene Organe und eine aktive Krankheitsmanifestation; 65 % der Teilnehmenden waren männlich; hinsichtlich der ethnischen Zusammensetzung waren 47 % asiatischer, 39 % weißer und 14 % anderer Herkunft. Bei 54 % der Patient:innen lag ein Krankheitsrezidiv vor; die mittlere Krankheitsdauer betrug 3,6 Jahre.

Alle Teilnehmenden erhielten in einem Zeitraum von 3 bis 8 Wochen vor Randomisierung eine Glukokortikoidtherapie. Zum Zeitpunkt der 1:1-Randomisierung gegen Placebo war die Prednisolon-Dosis auf 20 mg/Tag reduziert worden. Die Inebilizumab-Kohorte (*n* = 68) erhielt Inebilizumab in einer Dosierung von 300 mg intravenös an den Tagen 1 und 15 sowie erneut nach 26 Wochen. Die Glukokortikoiddosis wurde anschließend im 2‑Wochen-Rhythmus um 5 mg reduziert, sodass die Patient:innen nach 8 Wochen steroidfrei waren. Die Gabe weiterer immunsuppressiver Begleitmedikation war nicht zulässig; im Rezidivfall konnten jedoch erneut Glukokortikoide eingesetzt werden. Die Studiendauer betrug 52 Wochen. Primärer Studienendpunkt war die Zeit bis zum ersten behandlungspflichtigen Krankheitsschub. Sekundäre Endpunkte waren die jährliche Rate der Krankheitsschübe sowie die behandlungsfreie komplette Remission und glukokortikoidfreie komplette Remission nach 52 Wochen.

Im Ergebnis zeigte sich, dass die Behandlung mit Inebilizumab das Risiko von Krankheitsschüben signifikant verringerte: So wiesen nur 7 Studienteilnehmer:innen unter Inebilizumab-Therapie (10 %) einen Krankheitsschub auf, wohingegen es in der Placebokohorte 40 Teilnehmer:innen (60 %) waren. Die jährliche Schubrate war ebenfalls unter Inebilizumab signifikant niedriger als unter Placebo (*p* < 0,001) (vgl. Abb. 1).

**Abb. 1:**
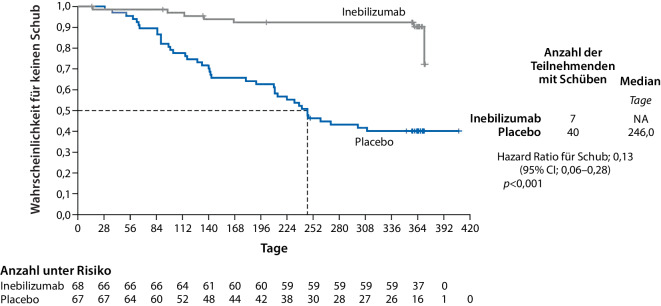
Zeit bis zum ersten Krankheitsrezidiv. *No. at Risk* „number at risk“, *CI* Konfidenzintervall. (Mod. nach [9])

Bezüglich der sekundären Endpunkte erreichten signifikant mehr Proband:innen in der Inebilizumab-Kohorte eine behandlungs- und schubfreie Komplettremission (Odds Ratio, 4,68; 95 % CI, 2,21–9,91; *p* < 0,001). Dies war ebenfalls im Hinblick auf eine schubfreie und glukokortikoidfreie Komplettremission (Odds Ratio, 4,96; 95 % CI, 2,34–10,52; *p* < 0,001) der Fall. Bezüglich relevanter Biomarker sah man, dass die IgG4-Spiegel im Schnitt nach 52 Wochen eine mediane Verringerung von 50 % zum Ausgangswert nach Inebilizumab-Therapie aufwiesen (Placebo ohne Änderung). Weiterhin zeigte sich eine anhaltende CD20 + B-Zell-Depletion über 52 Wochen unter Inebilizumab-Therapie.

Hinsichtlich der Sicherheitsdaten ist herauszustellen, dass die Anzahl der schwerwiegenden unerwünschten Ereignisse in der Inebilizumab-Kohorte doppelt so hoch wie in der Placebokohorte war (*n* = 12 [18 %] vs. *n* = 6 [9 %]). Die häufigsten unerwünschten Ereignisse waren COVID-Infektionen (*n* = 16; 24 %), Lymphopenien (*n* = 11; 16 %) und Harnwegsinfektionen (*n* = 8; 12 %). Todesfälle traten nicht auf.

Zusammenfassend belegt die MITIGATE-Studie, dass eine CD19-gerichtete B‑Zell-Therapie mit Inebilizumab bei der Behandlung der IgG4-RD wirksam eingesetzt werden kann. Die Ergebnisse untermauern die Rationale einer B‑Zell-zielgerichteten Therapie bei dieser Erkrankung. Offen bleibt jedoch die Frage, ob sich durch die Behandlung eine anhaltende, steroidfreie Krankheitsremission über einen Zeitraum von mehr als 52 Wochen erzielen lässt oder ob eine fortgesetzte Therapie mit Inebilizumab zum Remissionserhalt erforderlich ist. Zur Klärung dieser Fragestellung sind dringend Langzeitdaten notwendig.

Neben dem Einsatz monoklonaler Antikörper gegen CD19 zeichnet sich in der Rheumatologie derzeit eine „zweite therapeutische Revolution“ ab: Durch den erfolgreichen Einsatz von gegen CD19 gerichteten CAR-T-Zell-Therapien [11] sowie bispezifischen T‑Zell-Engagern wie Blinatumomab [12, 13] eröffnen sich neue Behandlungsoptionen für systemische, B‑ und Plasmazell-vermittelte Autoimmunerkrankungen. Vor diesem Hintergrund erscheint auch der therapeutische Einsatz dieser innovativen Ansätze bei der IgG4-RD als naheliegend. Jüngste präklinische Daten aus einem Mausmodell konnten bereits eine Wirksamkeit der Anti-CD19-CAR-T-Zell-Therapie bei IgG4-RD demonstrieren ([14]; vgl. Tab. 1).

**Tab. 1:** CD19-gerichtete Therapieoptionen bei IgG4-RD

Therapieoption	Wirkmechanismus	Evidenz bei IgG4-RD	Zulassungsstatus für IgG4-RD	Besonderheiten	Geschätzte Jahrestherapiekosten
Inebilizumab	Humanisierter monoklonaler Antikörper gegen CD19	Phase-3-Studie (MITIGATE) zeigt Wirksamkeit bei aktiver IgG4-RD [9]	*Zugelassen in den USA (04/2025)* *EMA noch ausstehend*	Erste zugelassene B‑Zell-Therapie bei IgG4-RD; 3 Dosen in Woche 0, 2 und 26	Ca. **420.745 USD/Jahr** (3 × 140.248,50 USD)
Obexelimab	Dualer Antikörper gegen CD19 und FcγRIIB (inhibitorischer Fc-Rezeptor)	Phase-2-Ergebnisse bei IgG4-RD mit positiver Wirkung [8]	*Nicht zugelassen*	Reduziert B‑Zell-Aktivierung ohne Depletion; potenziell niedrigere Infektionsraten möglich sowie Impfungen möglich [15]	Noch unbekannt
Rituximab	Monoklonaler Antikörper gegen CD20 (indirekt CD19)	Registerdaten und Open-Label-Studie mit klinischem Nutzen [6, 7]	*Nicht zugelassen*	„Off-label“ bei IgG4-RD; begrenzte Wirksamkeit auf Plasmazellen	Ca. **7000–14.000** **€** (abhängig von Dosis/Frequenz)
CD19-CAR-T-Zell-Therapie	Autologe T‑Zellen mit chimärem Antigenrezeptor gegen CD19	Präklinische Evidenz (Mausmodell) [14]	*Nicht zugelassen*	Potenziell kurativ, hohe Komplexität und höhere Toxizität der Therapie	Ca. **350.000–450.000** **€ **pro Behandlung** (einmalig)**
Blinatumomab	Bispezifischer T‑Zell-Engager (CD19 × CD3)	Bisher keine Daten für IgG4-RD	*Nicht zugelassen*	In anderen Indikationen etabliert (ALL); dort kontinuierliche Gabe nötig *Alternative (niedrigere) diskontinuierliche Dosierung in rheumatologischen Indikationen bisher verwendet [12, 13]	Ca. **100.000–300.000** **€ **(ALL-Therapie)

In den USA wurde Inebilizumab im April 2025 zur Therapie der IgG4-RD zugelassen; eine Zulassung durch die EMA wird zeitnah erwartet. Damit stünde erstmals eine spezifisch zugelassene Therapieoption für diese Erkrankung zur Verfügung. Vor diesem Hintergrund sollte Inebilizumab als neuer Therapiestandard für die Behandlung der IgG4-RD angesehen werden. Kritisch zu diskutieren ist hierbei allerdings die aktuelle Preisgestaltung: In den USA liegt der Listenpreis derzeit bei 140.248,50 USD pro Dosis [16]. Vor dem Hintergrund verfügbarer, jedoch bislang nicht zugelassener und deutlich kostengünstigerer Therapieoptionen wie z. B. MMF oder Rituximab – für die zumindest eine moderat gute Evidenzlage vorliegt – bleibt zu hoffen, dass die Preisgestaltung von Inebilizumab für Europa stärker im Einklang mit gesundheitsökonomischen Überlegungen erfolgt.
